# Effects of licorice crude extract on serum biochemistry, antioxidant capacity, immune function, rumen fermentation parameters, microbiota, and gastrointestinal enzyme activities in karakul sheep

**DOI:** 10.3389/fmicb.2026.1856043

**Published:** 2026-06-23

**Authors:** ShiJia Yang, RuiXuan Ji, Xin Ren, XueWen Chen, Abdul Shakoor Chaudhry, AnShan Shan, LianQun Wang, SuJiang Zhang

**Affiliations:** 1 Key Laboratory of Tarim Animal Husbandry Science and Technology, College of Animal Science and Technology, Tarim University, Alar, Xinjiang, China; 2 Key Laboratory of Livestock and Grass Resources Utilization Around Tarim, Ministry of Agriculture and Rural Areas (Co-construction by Ministries and Provinces), Tarim University, Alar, Xinjiang, China; 3 School of Agriculture, Food and Rural Development, Newcastle University, Newcastle upon Tyne, United Kingdom; 4 Institute of Animal Nutrition, Northeast Agricultural University, Heilongjiang, Harbin, China

**Keywords:** antioxidant capacity, immune function, licorice crude extract, rumen fermentation, sheep

## Abstract

This study aimed to determine the optimal dietary supplementation level of Licorice Crude Extract (LCE, derived from *Glycyrrhiza glabra*) by evaluating its effects on serum biochemistry, antioxidant capacity, immune function, rumen fermentation parameters, microbial community, and gastrointestinal enzyme activities in Karakul sheep. As a by-product of licorice processing, LCE is rich in bioactive components (e.g., flavonoids and polysaccharides) with potential antioxidant, ant-inflammatory, and rumen-modulating properties, making it a promising feed additive for ruminants. Thirty-two sheep with similar body weights were randomly divided into four groups and fed a basal diet supplemented with 0% (control), 1, 2, or 3% LCE for 75 days. The LCE supplementation significantly enhanced serum antioxidant capacity, humoral immunity, and rumen fermentation efficiency, improving systemic antioxidant capacity with a dose-dependent decrease in MDA (*P* < 0.05), and humoral immunity with increased immunoglobulins (*P* < 0.05) in sheep. These improvements occurred in alongside, a profound reshaping of the rumen microbiota. High-throughput sequencing revealed that LCE supplementation, particularly at 2%, significantly reshaped the microbial community structure (PCoA plot, *P* < 0.05) and enhanced α-diversity (e.g., increased Shannon index, *P* < 0.05). This restructuring was characterized by a marked increase in the abundance of the phylum *Firmicutes* and a significant reduction in the genus g_Prevotella at the 2% LCE level (*P* < 0.05). These microbial shifts were functionally consequential, driving significant improvements in the rumen environment such as elevated pH and increased total volatile fatty acids (tVFA) concentration (*P* < 0.05). Concomitantly, a significant increase in ruminal cellulase activity was observed (*P* < 0.05). In contrast, several fundamental serum metabolic parameters, including TP, BUN, and GLU, remained stable across all groups (*P* > 0.05). The most consistent and beneficial effects across the microbial and physiological parameters were observed at the 2% LCE supplementation. These findings reveal that dietary supplementation with LCE can improve the main physiological functions of sheep, with the most beneficial effects observed at the 2% inclusion level for promoting health and enhancing nutrient metabolic efficiency. This study provides a scientific basis for the utilization of LCE as a feasible feed additive in sheep production, contributing to the reduced farming costs and sustainable livestock development.

## 1 Introduction

Antibiotic feed additives have long been used in intensive livestock production to promote growth and prevent diseases (Zhang X. et al., 2022). However, their overuse has led to critical global concerns, including antimicrobial resistance, drug residues in animal products, and environmental contamination (Fu et al., 2014). While antibiotic bans address these risks, they often result in reduced growth performance and increased disease incidence in livestock, creating an urgent demand for safe, effective, and sustainable alternatives in animal nutrition. Chinese herbal medicines, characterized by their rich bioactive components and dual nutritional-pharmacological properties, have emerged as promising candidates to replace antibiotics (Tong et al., 2020).

Glycyrrhiza (*Glycyrrhiza* spp.), a perennial herb adapted to sunny and dry habitats, has high nutritional value, containing 12–16% crude protein, abundant amino acids and essential minerals (Marjhan, 2024). It is rich in various bioactive substances, such as glycyrrhizic acid, flavonoids, polysaccharides and proteins, which endow it with broad pharmacological effects including antimicrobial, antiviral, antioxidant, anti-inflammatory and immunomodulatory properties (Cao et al., 2022; Wang et al., 2015). The dried stems and leaves of licorice are palatable for livestock, and can be used as high-quality forage or supplementary feed in arid and semi-arid areas in winter and spring, and its powder can also improve the palatability of compound feeds (Singh et al., 2024; Varada et al., 2023; Yang et al., 2025). In recent years, *Glycyrrhiza uralensis* and its extracts have been widely used in livestock feed, which can improve growth performance, gastrointestinal health, antioxidant and immune capacity of livestock and poultry, and show good application potential in disease prevention (Dal Bosco et al., 2019; Jiang et al., 2020; Wang et al., 2022; You et al., 2023; Zhu et al., 2024).

Notably, industrial production of licorice extracts generates large amounts of LCE, a cost-effective and renewable by-product that retains high levels of proteins, flavonoids, and residual bioactive components (Dal Bosco et al., 2019). Despite the well-documented benefits of purified licorice extracts in pigs and rabbits, research on LCE as a feed additive in sheep remains scarce. The synergistic effects of LCE on rumen fermentation, intestinal enzyme activities, and microbial communities in sheep are still unclear, and the optimal supplementation level and related metabolic regulation mechanisms of LCE have not been fully elucidated, leaving a key research gap in this field.

Flavonoids in LCE can enhance antioxidant capacity by scavenging free radicals (Visavadiya et al., 2009), and its polysaccharides can regulate immune cell activity (Zhang J. et al., 2022). At the microbial level, LCE may affect the ratio of *Firmicutes* to *Bacteroidetes* in the rumen (Cholewińska et al., 2021), promote the growth of cellulolytic bacteria, and increase the production of volatile fatty acids, thereby improving nutrient utilization efficiency. In ruminants, licorice extracts have been reported to improve growth performance, antioxidant status, rumen fermentation, and nutrient utilization in beef cattle and sheep (Guo et al., 2019). Based on previous dose studies of licorice extracts in ruminants and the active component content of LCE, four supplemental levels (0, 1, 2, and 3%) were set in this study to screen the optimal dosage.

Most existing studies have focused on purified licorice extracts (e.g., flavonoids, glycyrrhizic acid) rather than industrial by-products. In contrast, the present study used LCE, a low-cost and renewable by-product of glycyrrhizic acid production. We comprehensively evaluated the effects of LCE on serum biochemistry, antioxidant capacity, immune function, rumen fermentation, gastrointestinal enzyme activities, and rumen microbiota in Karakul sheep, determined the optimal supplemental level (2%), and revealed its regulatory mechanism on rumen microbial structure and function. These findings fill the research gap of LCE application in sheep feeding, provide a scientific basis for using industrial by-products as green and safe antibiotic alternatives, reduce farming costs, and support the sustainable development of the livestock industry.

## 2 Materials and methods

### 2.1 Experimental site and collection of materials

This study was conducted in Alar, Xinjiang (40°30′N, 81°15′E). All experimental analyses were performed in the laboratories of Tarim University. Representative samples of LCE, were obtained from Xinjiang Xinnong Licorice Industry Co., Ltd., China. Representative samples of LCE were obtained from Xinjiang Xinnong Licorice Industry Co., Ltd., China. The LCE, a powdered by-product after the industrial extraction of glycyrrhizic acid, was characterized by its complex composition. Chemical analyses revealed that the LCE contained 2.85% ± 0.12% total flavonoids (quantified by UV spectrophotometry using rutin as the standard at 510 nm), 8.63% ± 0.35% total polysaccharides (extracted via ultrasonic-assisted water extraction and quantified by UV spectrophotometry using glucose as the standard at 490 nm; structural analysis performed via ion chromatography and high-performance size exclusion chromatography), 12.5% ± 0.8% organic acids, proteins, amino acids, and approximately 1.02% ± 0.05% residual glycyrrhizic acid (quantified using High-Performance Liquid Chromatography, HPLC).

### 2.2 Animals, diets, housing, and experimental design

A total of thirty-two healthy Karakul sheep (6–8 months old) of both sexes with similar initial body weight (BW, 26.50 ± 2.30 kg) were randomly allocated into four dietary treatment groups (*n* = 8 per group), balanced for sex, age and initial BW. Preliminary statistical analysis showed no significant differences between males and females for all measured parameters (*P* > 0.05). Therefore, sex was not included as a factor in the statistical model. Animals were randomly allocated to balance sex in each group (4 males and 4 females per group) to ensure uniform initial conditions. The basal diet (Table 1) was formulated with locally sourced ingredients to meet or exceed the nutritional requirements recommended by the Chinese Feeding Standards of Meat-Producing Sheep and Goats (Ministry of Agriculture and PRC, 2004). This diet was supplemented with 0, 1, 2, and 3% LCE for the corresponding groups of sheep. The experiment included a 15-day adaptation period to the diets and facilities, followed by a 60-day formal trial period for feeding and measurements. A completely randomized design was used to allocate diets to pre-determined groups of sheep.

**TABLE 1:** Composition and nutritional levels of the basal diet.

Ingredients	Content (%)	Nutrient	Nutrient levels (%)
Sweet sorghum silage	37	ME (MJ/kg)	16.21
Alfalfa	10	CP	16.45
Lawn grass	15	EE	4.229
Corn	18	NDF	69.49
Wheat bran	10	ADF	41.48
Soybean meal	5	Ca	1.40
Limestone	0.3	P	0.34
NaCl	0.7		
NaHCO_3_	1		
Premix^a^	3		
Total	100		

*^a^*The premix provided the following per kg of diets: Vitamin A (IU) = 9 750, Vitamin D3 (IU) = 2 450, Vitamin E (IU) = 19.5, nicotinic acid (mg) = 11.5, Fe (mg) = 66, Zn (mg) = 70, Mn (mg) = 38, S (mg) = 0.4, Se (mg) = 0.3, Ca (g) = 7.5, P (g) ≥ 0.75, NaCl (g) = 9.

However, the sheep were housed in individual stalls to ensure their exposure to the similar environment. Specifically, all sheep were reared under outdoor sheds (not in open outdoor areas or closed indoor houses). The individual stalls were made of metal frames, and the floor of the stalls was brick-paved and constructed at a slightly lower level than the surrounding ground to facilitate drainage and keep the stall dry. No additional bedding material was used on the brick floor during the entire experimental period. Throughout the study, all lambs were housed individually in the aforementioned stalls with *ad libitum* access to feed and water. The diets were provided twice daily at 10:00 and 19:00. The feeding environment was maintained at 5–15°C with a relative humidity of 40–60%. Feed intake of each sheep was recorded daily, and body weight was measured weekly to monitor growth performance.

### 2.3 Animal ethical code

This study was approved by the Science and Technology Ethics Committee of Tarim University (Approval No.: A702512002). All experimental procedures strictly adhered to the Chinese Guidelines for the Care and Use of Animals for Research (GB 14925, 2001) (Standardization Administration of China, 2001), as well as the provisions of the “Regulations on the Administration of Experimental Animals” and the “Guiding Opinions on the Humane Treatment of Experimental Animals” issued by the Ministry of Science and Technology of the People’s Republic of China.

The experimental researchers (Shijia Yang and Ruixuan Ji) have received professional training on animal experiment ethics and technical operations, mastering methods to minimize stress and pain in experimental sheep (such as standardized blood collection, gentle feeding management, and appropriate euthanasia procedures during slaughter). The animal feeding facilities, experimental design, and the number of experimental animals (32 sheep) were verified by the ethics committee as compliant with relevant standards. The sample size (*n* = 8 per group, total 32 sheep) was determined based on power analysis (power = 0.80, α = 0.05) and previous similar studies, to ensure statistical validity while minimizing the number of animals used. All efforts were made to reduce the number of experimental animals and avoid unnecessary harm to them.

### 2.4 Experimental termination and animal slaughter

After the 75-day experiment, all 32 experimental lambssheep were slaughtered following the standard operating procedures (SOPs) approved by the Animal Ethics Committee of Tarim University. Slaughter was conducted in the professional slaughterhouse affiliated with the university’s Animal Science and Technology College, adhering to humane euthanasia protocols prior to slaughter: lambssheep were anesthetized with an intravenous injection of sodium pentobarbital (50 mg/kg body weight) to minimize stress and pain, followed by exsanguination via jugular vein severance to ensure rapid and painless death.

#### 2.4.1 Blood sample collection and processing

Prior to slaughter (at 09:00 on the final day of the experiment), 10 mL of blood was collected from each sheep via the jugular vein using a sterile vacuum blood collection tube (without anticoagulant). The collected blood samples were centrifuged at 3,000 × g for 10 min at 4°C to separate serum. The supernatant serum was aspirated using a sterile pipette, transferred to sterile cryopreservation tubes, and immediately stored at −20°C for subsequent biochemical indicator detection.

#### 2.4.2 Rumen fluid (RF) collection and processing

Within 30 min post-slaughter, the abdominal cavity of each sheep was rapidly opened using sterile surgical instruments to expose the entire gastrointestinal tract. The rumen was carefully separated from other digestive organs (reticulum, omasum, abomasum, and intestines) by cutting the connecting mesentery to avoid cross-contamination. Approximately 20 mL of rumen fluid was collected from the ventral sac of the rumen using a sterile syringe; the collection site was selected to avoid excessive feed residues and ensure representative samples. The collected rumen fluid was immediately filtered through four layers of sterile gauze to remove solid particles, and the filtrate was used for immediate pH measurement with a calibrated digital pH meter. The abomasum is one of the four compartments of the sheep’s stomach (along with the rumen, reticulum, and omasum), and abomasal digesta were collected simultaneously with ruminal digesta post-slaughter for subsequent enzyme activity detection. The remaining rumen fluid and other gastrointestinal contents were retained in sterile cryopreservation containers for subsequent nutrient and microbial analysis. All rumen fluid samples were immediately snap-frozen in liquid nitrogen and stored at −80°C.

#### 2.4.3 Intestinal tissue and mucosa collection and processing

After collecting rumen fluid, the entire intestinal tract was further separated and fixed to facilitate tissue sampling. Segments (approximately 5 cm in length) were excised from the mid-jejunum, mid-ileum, and mid-cecum using sterile scissors and forceps, ensuring that the sampled segments were free of damage, inflammation, or abnormal lesions. Each intestinal segment was gently rinsed with pre-cooled (4°C) sterile physiological saline to remove intestinal contents; the physiological saline used was 0.9% (w/v) sodium chloride solution (NaCl concentration: 9 g/L, pH 7.2–7.4, sterile filtered through a 0.22 μm filter membrane to ensure sterility and avoid microbial contamination). After rinsing, the mucosal layer of each intestinal segment was carefully scraped off using a sterile glass slide, collected into sterile cryopreservation tubes. All collected intestinal tissue segments and mucosal samples were immediately snap-frozen in dry ice to preserve their biological activity, and then transferred to a −80°C ultra-low temperature freezer for long-term storage. For 16S rDNA sequencing analysis (to detect microbial community composition), a representative subset of samples was selected. Specifically, three sheep per group (12 sheep in total) were randomly selected based on the group mean values of body weight, feed intake, and key serum indicators (immunoglobulin G, IgG; malondialdehyde, MDA), ensuring that the selected samples were representative of the overall growth performance and physiological status of each treatment group.

### 2.5 Measurements and analysis

#### 2.5.1 Analysis of blood serum

Serum samples were thawed at 4°C, analyzed using an Automatic Biochemical Analyzer (AU480; Beckman Coulter, Brea, CA, United States) for total protein (TP), albumin (ALB), globulin (GLB), total cholesterol (TC), triglyceride (TG), blood urea nitrogen (BUN), glucose (GLU), alkaline phosphatase (ALP), and alanine aminotransferase (ALT). the activities of glutathione peroxidase (GSH-Px) and superoxide dismutase (SOD), the concentration of malondialdehyde (MDA), the total antioxidant capacity (T-AOC), and the levels of immunoglobulin A (IgA), immunoglobulin G (IgG), and immunoglobulin M (IgM) by using relevant commercial ELISA kits (Shanghai Enzyme-linked Biotechnology Co., Ltd., China) and a microplate reader (Guo et al., 2019).

#### 2.5.2 Analysis of rumen fluid

The Ammonia–nitrogen (NH_3_-N) was analyzed by the phenol-hypochlorite reaction according to the steps outlined by Weatherburn (1967). Rumen liquid (10 mL) was mixed with 2.5 mL of a HPO3 (250 g/L), and then centrifuged at 4°C for 10 min (12,000 × g) for total volatile fatty acids (tVFA) analysis. The rumen tVFA (i.e., acetate, propionate, butyrate) analysis was performed by gas chromatography (GC-2014FRGA1, Shimadzu, Tokyo, Japan). The injector temperature was 250°C while the oven and the detector were maintained at 160°C. Nitrogen was used as both the carrier gas (flow rate 0.6 mL/min) and the make-up gas (flow rate 25 mL/min). The flowrates of hydrogen and air were 20 and 300 mL/min, respectively (Julák et al., 2000).

#### 2.5.3 Digestive enzyme activity

The activities of cellulase, neutral protease, and α-amylase were measured in ruminal and abomasal digesta, while the activities of trypsin, cellulase, lipase, and α-amylase were assessed in the duodenum, jejunum, and ileum. Each sample underwent a homogenization pretreatment using a specific homogenization medium: 0.9% (w/v) sterile normal saline (i.e., 9 g/L sodium chloride solution, pH 7.2–7.4, sterile filtered through a 0.22 μm filter membrane to avoid microbial contamination and enzyme inactivation). Specifically, 1 g of frozen digesta sample (ruminal, abomasal, duodenal, jejunal, or ileal) was accurately weighed and added to the aforementioned homogenization medium at a mass-volume ratio of 1:9 (g/mL). Mixing took place in an ice water bath to prepare a 10% homogenate, which helped maintain enzyme activity and prevent denaturation. The homogenate was then centrifuged at 4,000 rpm for 10 min at room temperature, the supernatant was collected, and the target enzyme activity was determined according to the instructions of the appropriate kits. Cellulase, neutral protease, lipase, α-amylase, and trypsin activities were measured using detection kits (Nanjing Jiancheng Bioengineering Institute, Nanjing, China; catalog numbers: A007-1 for cellulase, A080-1 for neutral protease, A054-1 for lipase, A003-1 for α-amylase, A080-2 for trypsin) following the manufacturers’ instructions. The enzyme activity units were defined as follows: one unit (U) of cellulase activity corresponds to the amount of enzyme that releases 1 μmol of glucose per gram of sample per minute at 37°C and pH 7.0; one unit of neutral protease activity is the amount of enzyme that releases 1 μmoL of tyrosine per gram of sample per minute at 37°C and pH 7.5; one unit of lipase activity is the amount of enzyme that releases 1 μmol of fatty acid per gram of sample per minute at 37°C and pH 7.5; one unit of α-amylase activity is the amount of enzyme that hydrolyzes 1 mg of starch per gram of sample per minute at 37°C and pH 6.9; one unit of trypsin activity is the amount of enzyme that releases 1 μmol of tyrosine per gram of sample per minute at 37°C and pH 8.0. All assays were performed in triplicate.

#### 2.5.4 DNA extraction, 16S rDNA gene amplification, sequence processing and analysis

The DNA samples of Rumen fluid were directly extracted with TIANamp Stool DNA Kit (TIANGEN, Beijing, China) following the manufacturer’s protocol. The *V3–V4* hypervariable region of the bacterial *16S rRNA* gene were amplified by PCR using primers 338F (5′-ACTCCTACGGGAGGCAGCAG-3′) and 806R (5′-GGACTACHVGGGTWTCTAAT-3′), which were verified for specificity to bacterial 16S rDNA (synthesized by Shanghai Sangon Biological Engineering Co., Ltd., China) (Sun et al., 2019). The total volume of the reaction mixture was 50 μL, which consisted of 0.35 μg of template DNA, 2 μL primer mix (10 μM), 5μL 10 × Taq Buffer (TIANGEN, Beijing, China), 4 μL dNTP mixture (2.5 mM), 0.5 μL DNA polymerase (2.5 U/μL, TIANGEN, Beijing, China), and approximately 38.5 μL milli-Q water. Thermocycling parameters were as follows: initial denaturation at 95°C for 5 min; 35 cycles of denaturation at 95°C for 30 s, annealing at 50°C for 30 s, and extension at 72°C for 90 s; and final extension at 72°C for 10 min. PCR products were verified by 1.5% agarose gel electrophoresis, and three amplicons per group were selected and sent to Novogene Technology Co. (Beijing, China) for sequencing on an Illumina MiSeq PE300 platform according to the standard procedures.

Raw reads were filtered to obtain the final clean reads using FASTP (Chen et al., 2018), then the noisy sequences of raw tags were analyzed using QIIME (version V1.9.1) (Caporaso et al., 2010). After removal of chimeric sequences with VSEARCH (Rognes et al., 2016), all clean reads were clustered into operational taxonomic units (*OTU*s) using Uparse software (Edgar, 2013) at a similarity of 97%. The most abundant sequences in *OTU*s were screened out as the representative sequences by the Silva database 132 (Quast et al., 2013), according to the reference taxonomy provided by the SSU rRNA database. Five alpha indices including Observed species, Shannon, Simpson, Chao1, and ACE were calculated. Principal coordinates analysis (PCoA) based on Bray–Curtis distance was estimated to reveal beta diversity in bacterial communities among the four groups. The bacterial compositions of each group at phylum and genus level were conducted using OriginPro software (version 9.0) to obtain relative abundance histograms.

### 2.6 Statistical analysis

All statistical analyses were performed using SPSS software with a significance level of *P* < 0.05. The resultant data were shown as means ± standard errors (*n* = 3). One-way ANOVA was used to analyze the effects of different LCE supplementation levels (0, 1, 2, 3%) on growth performance, serum indicators, rumen fermentation parameters, digestive enzyme activities, and rumen/intestinal microbiome alpha diversity. Repeated-measures ANOVA was applied to data, with Duncan’s *post-hoc* test for multiple comparisons among groups. Polynomial regression analysis was used to explore LCE dose-effect relationships, while microbiome data were further analyzed by PCoA, PERMANOVA, and LEfSe using QIIME 2 software to determine differences in microbial community structure and composition. Sex was not included as a source of variation because no significant sex effects were observed for any parameter (*P* > 0.05).

## 3 Results

### 3.1 Physiological status in blood

As shown in the statistical results in Table 2, the supplementation of LCE had a significant impact on certain serum biochemical parameters in meat sheep. Specifically, The albumin (ALB) level in the 0% group was significantly higher than that in the 2 and 3% groups (*P* < 0.05), with no significant difference between the 0 and 1% groups. The GLB in the 2% group was significantly higher than that in the 0 and 1% groups (*P* < 0.05). The ALT activity in the 1% group was significantly higher than that in the 2% group (*P* < 0.05), with no significant differences among the 0, 1, and 3% groups. The A/G ratio in the 0 and 1% groups was significantly higher than that in the 2 and 3% groups (*P* < 0.05), consistent with the changes in ALB. The TC level in the 0% group was significantly higher than that in the 2% group (*P* < 0.05), with no significant differences among the 0, 1, and 3% groups. LCE supplementation significantly affected BUN and GLU (*P* < 0.05), but had no significant effects on TP, ALP, or TG (*P* > 0.05), indicating overall metabolic stability.

**TABLE 2:** Effects of adding LCE on blood serum indices of sheep.

Items	Groups				*P*-value
	0%	1%	2%	3%	
ALB/μg/mL	34.93 ± 0.15a	34.33 ± 0.70ab	33.77 ± 0.75b	33.23 ± 0.75b	<0.001
GLB/μg/mL	24.03 ± 1.42ab	23.36 ± 0.54c	25.35 ± 0.82a	24.61 ± 1.78ab	<0.001
TP/μg/mL	58.30 ± 0.87	57.73 ± 1.23	58.87 ± 1.04	58.57 ± 1.85	0.891
A/G	1.46 ± 0.08ab	1.47 ± 0.02a	1.33 ± 0.06c	1.35 ± 0.07ab	0.0015
BUN/mg/mL	3.27 ± 0.36ab	3.12 ± 0.42ab	3.01 ± 0.27c	3.37 ± 0.32a	<0.001
ALT/ng/L	28.67 ± 1.10ab	30.17 ± 1.76a	28.33 ± 1.53c	28.67 ± 2.03ab	0.028
ALP/ng/L	110.00 ± 3.40	122.00 ± 3.72	155.00 ± 7.09	115.67 ± 2.35	0.576
TG/mg/mL	0.18 ± 0.05ab	0.20 ± 0.07a	0.13 ± 0.05c	0.17 ± 0.04ab	0.453
TC/μg/mL	1.77 ± 0.13a	1.63 ± 0.05ab	1.60 ± 0.06c	1.67 ± 0.08ab	0.048
GLU/mg/mL	4.47 ± 0.23a	4.13 ± 0.06ab	4.03 ± 0.16c	4.07 ± 0.17ab	< 0.001

ALB, albumin; GLB, globulin; TP, total protein; A/G, albumin-to-globulin ratio; BUN, blood urea nitrogen; ALT, alanine aminotransferase; ALP, alkaline phosphatase; TG, triglyceride; TC, total cholesterol; GLU, glucose. In the same row, values with no letter or the same letter superscripts are not significantly different (*P* > 0.05), whereas those with different small letter superscripts are significantly different (*P* < 0.05).

As presented in Table 3, the serum MDA content decreased significantly in a dose-dependent manner with increasing LCE supplementation (*P* < 0.05). The activity of GSH-Px was significantly elevated in the 1% LCE group compared to all other groups (*P* < 0.05). In contrast, no significant differences were observed in T-AOC or SOD activities among the groups (*P* > 0.05). However, statistical analysis revealed no significant differences in IgG, IgM, and IgA levels among groups (*P* > 0.05), indicating that the addition of crude glycyrrhiza extract had no significant effect on serum immunoglobulin levels in sheep, although a certain positive upward trend was observed. Significant differences in interleukin-1β (IL-1β) levels were observed among groups (*P* < 0.05), with the highest concentration detected in the 3% addition group, showing statistically significant differences compared to the control group and other treatment groups. Similarly, significant intergroup differences were observed for tumor necrosis factor-α (TNF-α) (*P* < 0.05), with the highest concentration recorded in the 1% addition group, markedly higher than that in the control group. No statistically significant differences were found for interleukin-2 (IL-2) and interleukin-4 (IL-4) across groups (*P* > 0.05).

**TABLE 3:** Effect of adding LCE on antioxidant and immune indeces of serum in sheep.

Items	Groups				*P*-value
	0%	1%	2%	3%	
SOD (U/mL)	4.67 ± 0.33c	4.76 ± 0.49a	4.75 ± 0.22bc	4.69 ± 0.32bc	0.87
MDA/nmol/mL	0.89 ± 0.04a	0.75 ± 0.05bc	0.77 ± 0.05bc	0.62 ± 0.06c	<0.001
GSH-Px/pmol/mL	139.52 ± 5.62bc	165.50 ± 8.05a	144.65 ± 3.23bc	136.72 ± 7.74c	<0.001
T-AOC/μmol/mL	0.95 ± 0.04	0.98 ± 0.04	0.96 ± 0.02	0.99 ± 0.05	0.76
IgG	20.67 ± 2.37	25.68 ± 0.97	30.10 ± 19.24	31.37 ± 13.01	0.215
IgM	25.07 ± 4.80	34.85 ± 4.90	29.58 ± 5.98	26.93 ± 9.35	0.128
IgA	18.25 ± 9.64	36.32 ± 6.95	32.66 ± 6.52	42.10 ± 5.87	0.201
IL-1β	32.17 ± 5.06b	30.03 ± 4.72b	36.15 ± 4.01b	53.88 ± 9.38a	0.005
IL-2	22.91 ± 6.31	25.24 ± 7.51	28.65 ± 10.81	39.02 ± 7.85a	0.115
IL-4	35.20 ± 1.61	39.21 ± 7.66	37.16 ± 0.83	38.17 ± 3.76	0.718
TNF-α	30.18 ± 3.46c	47.98 ± 4.87a	42.30 ± 5.22ab	43.28 ± 5.00ab	0.013

In the same row, values with no letter or the same letter superscripts are not significantly different (*P* > 0.05), whereas those with different small letter superscripts are significantly different (*P* < 0.05).

### 3.2 Rumen pH and volatile fatty acid concentration

Table 4 illustrates the effects of LCE supplementation on ruminal pH, NH_3_-N concentration, and tVFA levels in meat sheep. The ruminal pH was significantly higher in the 3% group than in the 0 and 1% groups (*P* < 0.05), with no significant difference between the 2 and 3% groups or between the 0 and 1% groups. Although the NH_3_-N content did not differ significantly among groups with increasing supplementation levels (*P* > 0.05), a general decreasing trend of NH_3_-N content was noted in the treatment groups. The acetate (AA), butyrate (BA), and total VFA concentrations in the 2% group were significantly higher than those in the 0, 1, and 3% groups (*P* < 0.05).

**TABLE 4:** Effect of adding LCE on rumen pH and volatile fatty acid concentration in sheep.

Items	Groups				*P*-value
	0%	1%	2%	3%	
pH	6.42 ± 0.14bc	6.40 ± 0.16c	6.65 ± 0.15bc	6.69 ± 0.20a	0.021
NH_3_-N, mg/mL	10.13 ± 3.47	8.51 ± 1.61	8.19 ± 1.89	7.31 ± 1.96	0.53
Acetate, mmol/L	54.01 ± 1.54c	58.42 ± 0.39bc	71.54 ± 4.76a	61.88 ± 7.08bc	<0.001
Propionate, mmol/L	24.05 ± 0.63a	22.19 ± 0.19bc	19.89 ± 0.33bc	17.25 ± 0.31c	0.011
Butyrate, mmol/L	11.12 ± 1.11c	13.58 ± 0.72bc	22.31 ± 0.89bc	19.15 ± 0.30a	<0.001
Total volatile fatty acid, mmol/L	89.18 ± 2.91c	94.19 ± 0.32bc	113.74 ± 4.51a	98.29 ± 1.02bc	<0.001

LA, lactic acid; AA, acetic acid; BA, butyric acid; NH_3_-N, ammonia nitrogen. In the same row, values with no letter or the same letter superscripts are not significantly different (*P* > 0.05), whereas those with different small letter superscripts are significantly different (*P* < 0.05).

### 3.3 Digestive enzyme activity

As shown in Table 5, dietary LCE supplementation significantly altered digestive enzyme activities across various gastrointestinal segments of sheep (*P* < 0.05). In the rumen, neutral protease activity was significantly higher in the control (0% LCE) group than in the supplemented groups (*P* < 0.05). In contrast, the activities of α-amylase and cellulase increased significantly with the rising LCE levels (*P* < 0.05), reaching their peaks at 3% supplementation, respectively. In the abomasum, neutral protease activity was significantly elevated in the 1% LCE group compared to others (*P* < 0.05), while α-amylase activity was concurrently suppressed (*P* < 0.05). Abomasal cellulase activity differed significantly among groups (*P* < 0.05), with the highest value observed in the 2% group. Within the duodenum, significant variations were noted: trypsin activity was higher in the 1 and 3 groups (*P* < 0.05), α-amylase activity peaked in the 1% group (*P* < 0.05), and both cellulase and lipase activities were greatest in the 3% group (*P* < 0.05). In the jejunum, α-amylase and cellulase activities were significantly higher in the control group than in LCE-supplemented groups (*P* < 0.05), whereas lipase activity was significantly increased in the 3% group (*P* < 0.05). The most complex response patterns were observed in the ileum: trypsin activity was highest in the 1% group (*P* < 0.05), α-amylase peaked in the 2% group (*P* < 0.05), and in the ileum, lipase activity was significantly higher in the 2% group than in other groups (*P* < 0.05). It is noteworthy that all enzymatic activities in the ileum remained significantly lower than those in the foregut (*P* < 0.05).

**TABLE 5:** Effect of adding LCE on ruminal and intestinal enzyme activity in sheep (U/g fresh weight).

Items	Groups				*P*-value
	0%	1%	2%	3%	
Rumen					
Neutral protease	9.24 ± 0.42a	2.70 ± 0.05c	4.21 ± 0.26bc	2.89 ± 0.50c	< 0.01
α-amylase	602.21 ± 26.22b	475.53 ± 14.38c	944.27 ± 37.23b	1133.22 ± 51.06a	< 0.01
Cellulase	1379.30 ± 57.06c	1304.15 ± 57.93c	1541.11 ± 96.76b	1547.41 ± 119.80a	< 0.01
Abomasum					
Neutral protease	4.97 ± 0.41c	27.34 ± 2.22a	6.20 ± 0.56bc	7.84 ± 0.43b	< 0.01
α-amylase	424.96 ± 18.28a	273.56 ± 6.02b	199.32 ± 4.18c	242.93 ± 3.50b	< 0.01
Cellulase	1388.20 ± 123.83b	1322.40 ± 49.16c	1416.78 ± 36.13a	1390.50 ± 129.39b	< 0.01
Duodenum					
Trypsin	79.62 ± 7.70c	121.37 ± 6.44a	95.05 ± 8.14b	120.52 ± 0.97a	< 0.01
α-amylase	246.38 ± 5.85b	341.81 ± 12.08a	221.34 ± 3.35c	256.37 ± 3.50b	< 0.01
Cellulase	1311.98 ± 62.08c	1471.69 ± 78.97a	1408.31 ± 79.94b	1528.89 ± 68.51a	< 0.01
Lipase	153.39 ± 14.52b	143.96 ± 2.18b	97.48 ± 5.84c	185.40 ± 3.92a	< 0.01
Jejunum					
Trypsin	93.44 ± 5.22ab	88.14 ± 8.26b	91.80 ± 4.32ab	94.47 ± 4.80a	< 0.01
α-amylase	315.97 ± 5.07a	260.06 ± 2.34b	230.35 ± 5.97b	198.97 ± 8.97c	< 0.01
Cellulase	1384.37 ± 69.83a	1371.01 ± 57.37ab	1348.66 ± 32.90b	1284.03 ± 59.38c	< 0.01
Lipase	166.10 ± 4.90b	141.28 ± 3.19b	106.77 ± 3.57c	216.86 ± 6.13a	< 0.01
Ileum					
Trypsin	66.61 ± 6.43b	81.91 ± 2.42a	48.70 ± 2.20c	27.09 ± 2.68d	< 0.01
α-amylase	169.86 ± 6.74c	301.69 ± 6.86b	430.86 ± 9.46a	279.04 ± 6.06b	< 0.01
Cellulase	1346.75 ± 34.36a	1296.31 ± 16.40b	1256.67 ± 27.33c	1282.64 ± 4.28b	< 0.01
Lipase	67.26 ± 0.58c	68.70 ± 3.93bc	79.35 ± 6.93a	69.36 ± 3.14b	< 0.01

In the same row, values with no letter or the same letter superscripts are not significantly different (*P* > 0.05), whereas those with different small letter superscripts are significantly different (*P* < 0.05).

### 3.4 Diversity of rumen microbes

#### 3.4.1 Venn diagram analysis of ruminal microbiota

**FIGURE 1:**
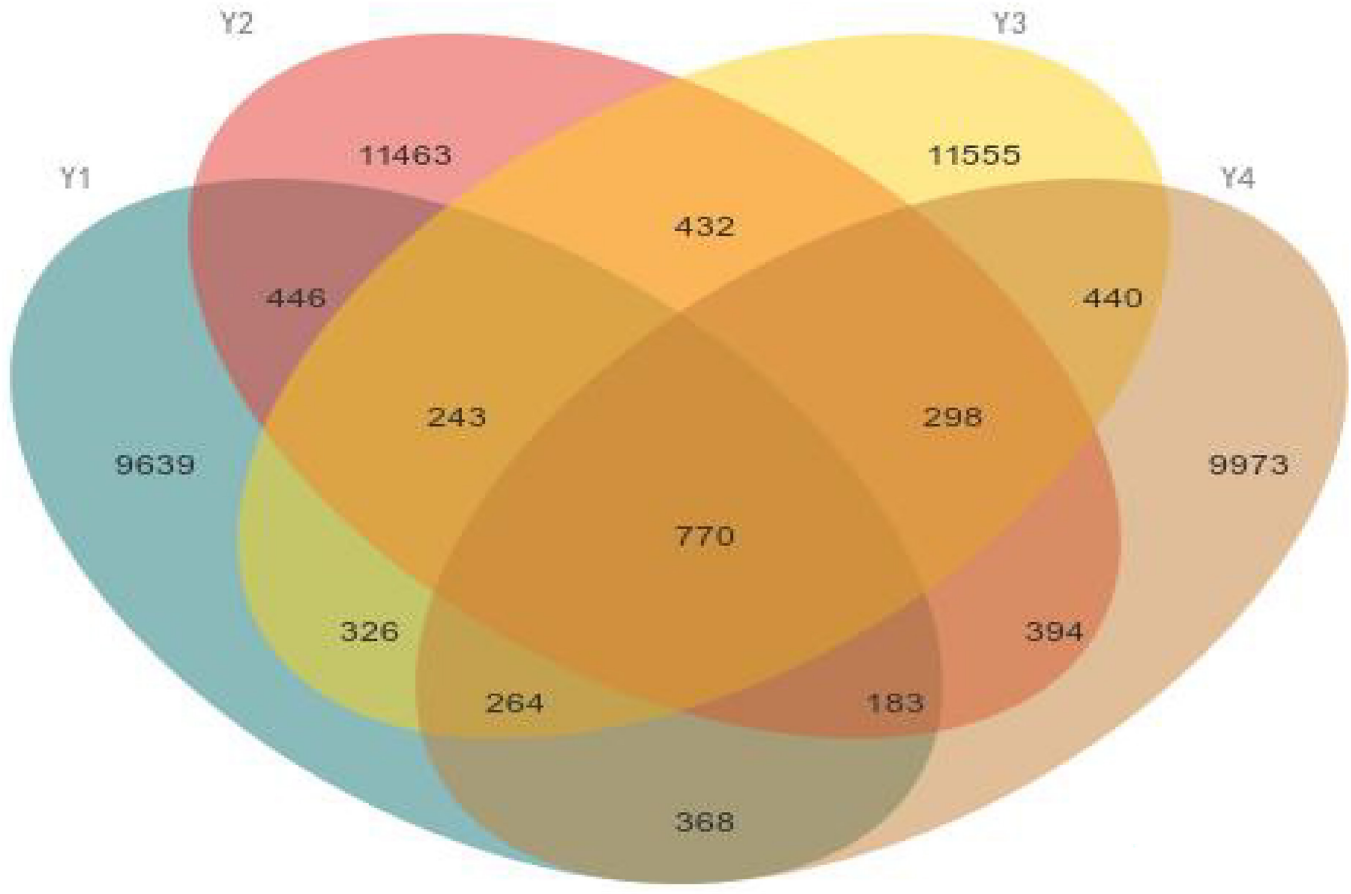
Venn diagram of *OTU*s in the rumen microbiota of sheep fed different levels of LCE supplementation. The overlapping region represents shared *OTU*s among groups, and the non-overlapping regions represent group-specific unique *OTU*s. Y1, 0% LCE; Y2, 1% LCE; Y3, 2% LCE; Y4, 3% LCE.)

As illustrated in Figure 1, the Venn diagram depicts the distribution of shared and unique operational taxonomic units (*OTU*s) among the four LCE supplementation groups. A total of 770 *OTU*s were shared across all groups, representing the core rumen microbial taxa conserved regardless of dietary LCE addition. The number of unique *OTU*s varied among groups: 9,639 in the 0% LCE group (Y1), 11,463 in the 1% LCE group (Y2), 11,555 in the 2% LCE group (Y3), and 9,973 in the 3% LCE group (Y4). Although the differences in unique *OTU* counts between Y2 and Y3 were not statistically significant (*P* > 0.05), both groups exhibited significantly higher values compared to Y1 and Y4 (*P* < 0.05), indicating that the groups with a greater number of unique taxa, such as Y2 and Y3, demonstrated relatively richer microbial diversity.

#### 3.4.2 Alpha diversity analysis of ruminal microbiota

**FIGURE 2:**
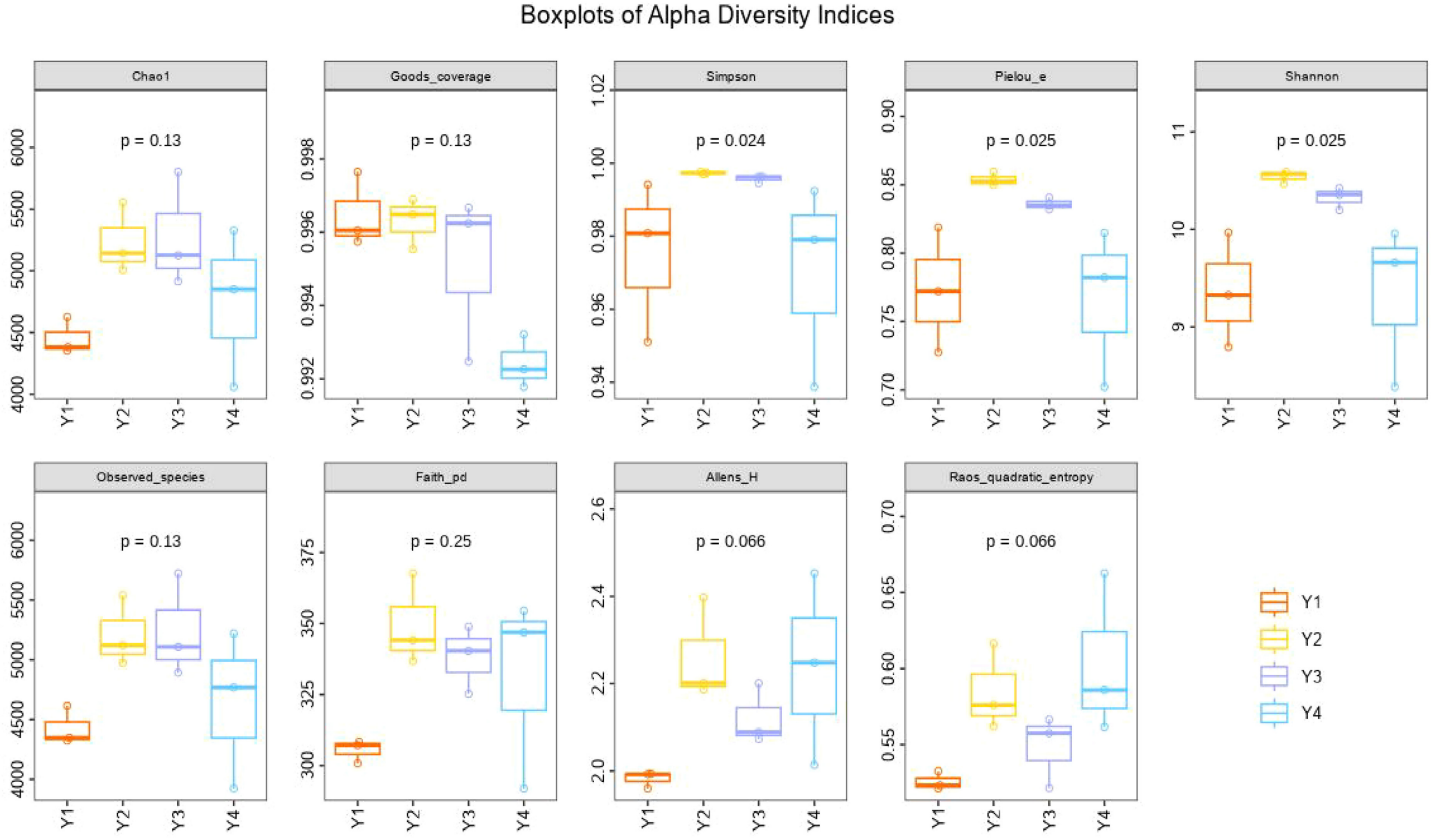
Box plot of differences between groups for alpha diversity analysis (Y1-Y4 = 0, 1, 2, 3% LCE groups, respectively).

**FIGURE 3:**
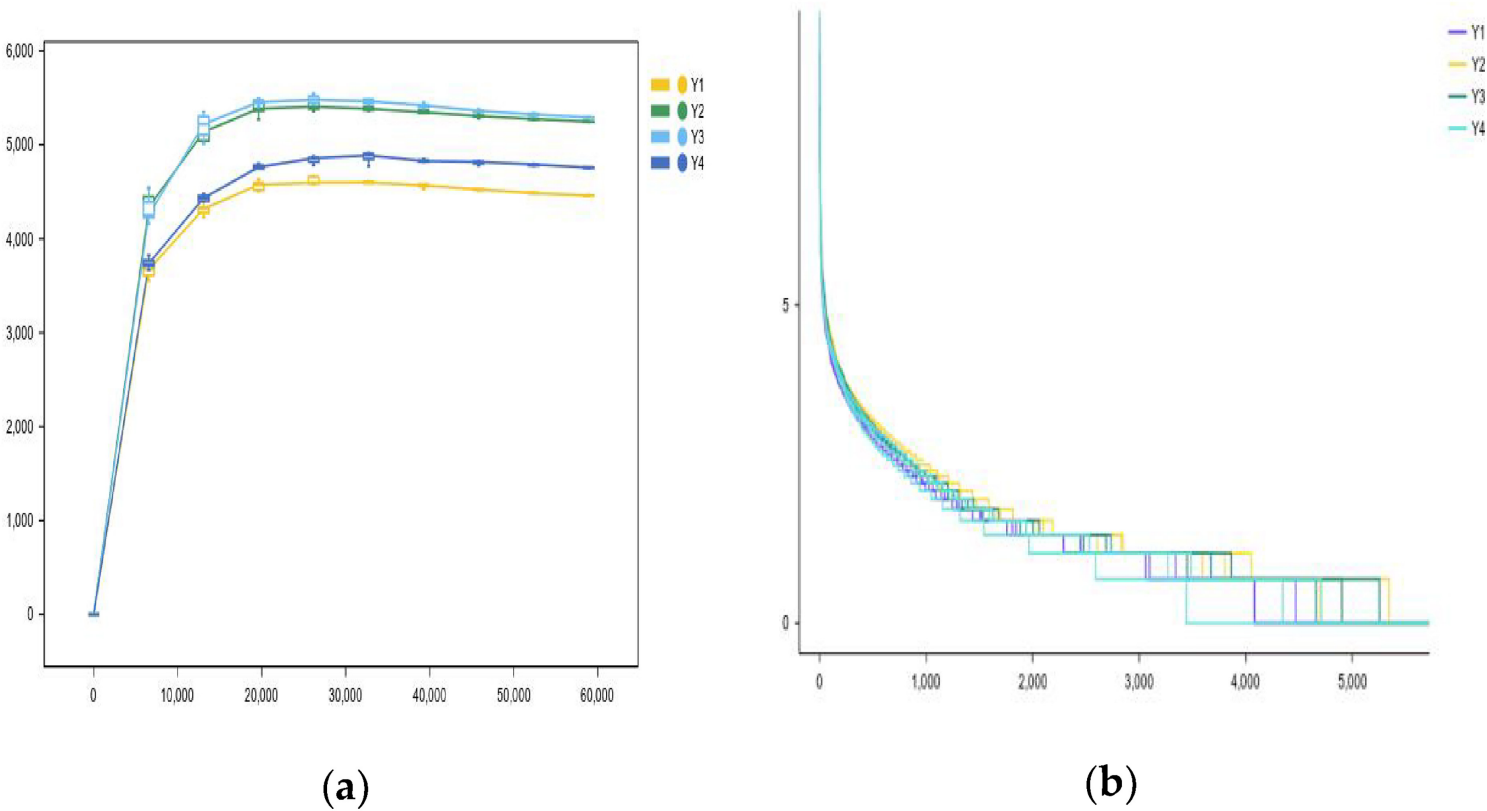
Rarefaction curves **(a)** and rank-abundance curves **(b)** of rumen microbiota in different groups.

As presented in Figure 2 (Box plot of alpha diversity differences), Figure 3 (Rarefaction curves and Rank-abundance curves), the alpha diversity of rumen microbiota among different LCE supplementation groups was comprehensively evaluated using rarefaction curves, rank-abundance curves, and diversity indices: rarefaction curves (Figure 3a) approached saturation at ∼20,000 sequencing reads, confirming sufficient sequencing depth and higher observed *OTU* numbers in the 1% (Y2) and 2% (Y3) LCE groups than in the 0% (Y1) and 3% (Y4) groups; rank-abundance curves (Figure 3b) showed longer curves (higher richness) for Y2 and Y3 and the flattest curve (better evenness) for Y3, while Y1 and Y4 had steeper curves indicating more dominant species; alpha diversity index analysis revealed significant differences in Simpson, Pielou_e, and Shannon indices (*P* < 0.05), with the highest Simpson and Pielou_e in Y2, the highest Shannon index in Y3 (significantly higher than Y1’s, *P* < 0.05), significantly higher Faith’s phylogenetic diversity in Y2 and Y3 than in Y1 (*P* < 0.05), and higher Allen’s H index and Rao’s quadratic entropy in all LCE-supplemented groups than in Y1 (*P* < 0.05), collectively demonstrating that the 1 and 2% LCE supplementation enhances rumen microbial richness, evenness, and phylogenetic diversity, with Y3 showing optimal comprehensive diversity.

#### 3.4.3 Beta diversity analysis (PCoA and hierarchical clustering)

**FIGURE 4:**
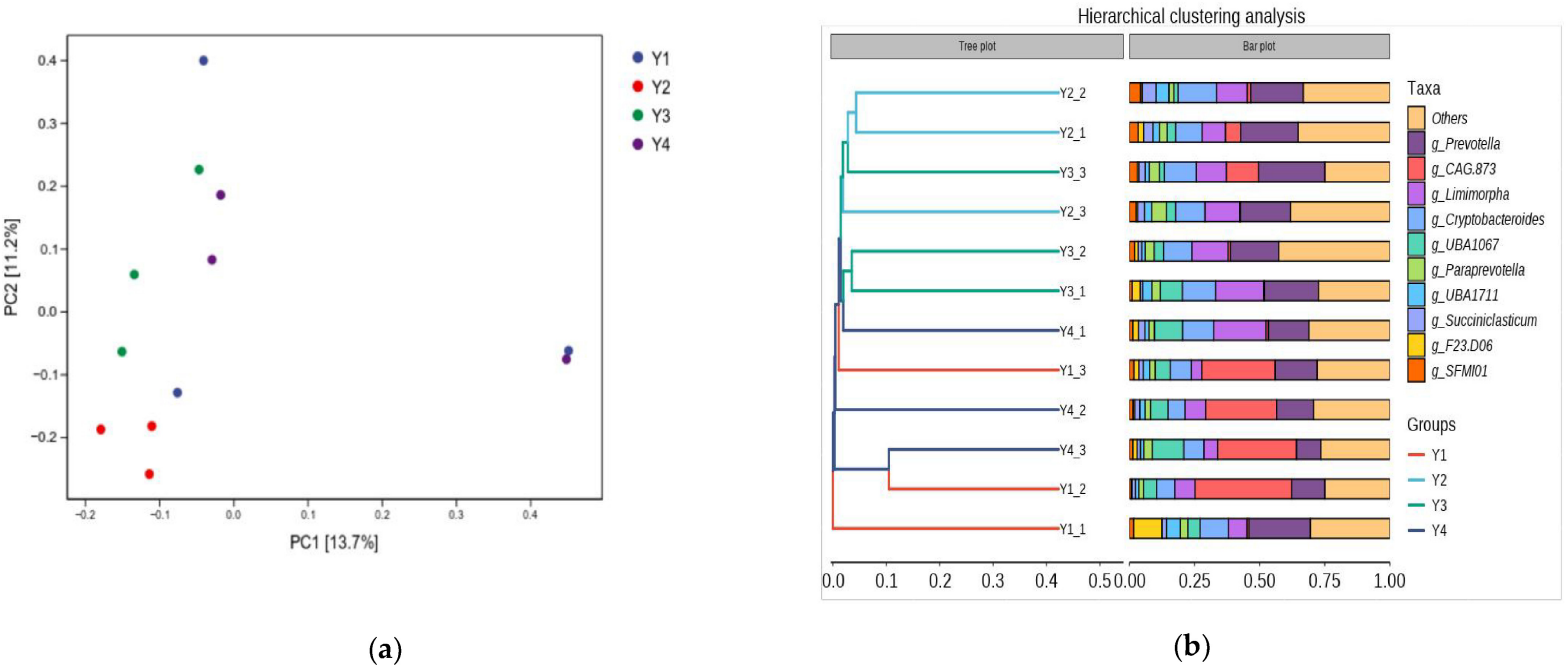
Analysis of rumen microbiota beta diversity. **(a)** PCoA plot based on Bray–Curtis distance. **(b)** Hierarchical clustering analysis, Y1, Y2, Y3, and Y4 represent the 0, 1, 2, and 3% LCE groups, respectively).

As presented in Figure 4, the beta diversity of rumen microbiota was evaluated using PCoA and hierarchical clustering analysis. The PCoA plot based on Bray–Curtis distance showed a clear separation of microbial communities among the four LCE supplementation groups (*P* = 0.001), indicating distinct differences in community structure between groups. Hierarchical clustering analysis further revealed that the samples within the same group were clustered closely together, while samples from different groups were separated by large branch distances. No significant differences were observed in the microbial composition among intra-group samples (*P* > 0.05), whereas inter-group differences were statistically significant (*P* < 0.05).

#### 3.4.4 Visualization of ruminal microbial taxonomic hierarchy and distribution patterns based on Sankey diagram

**FIGURE 5:**
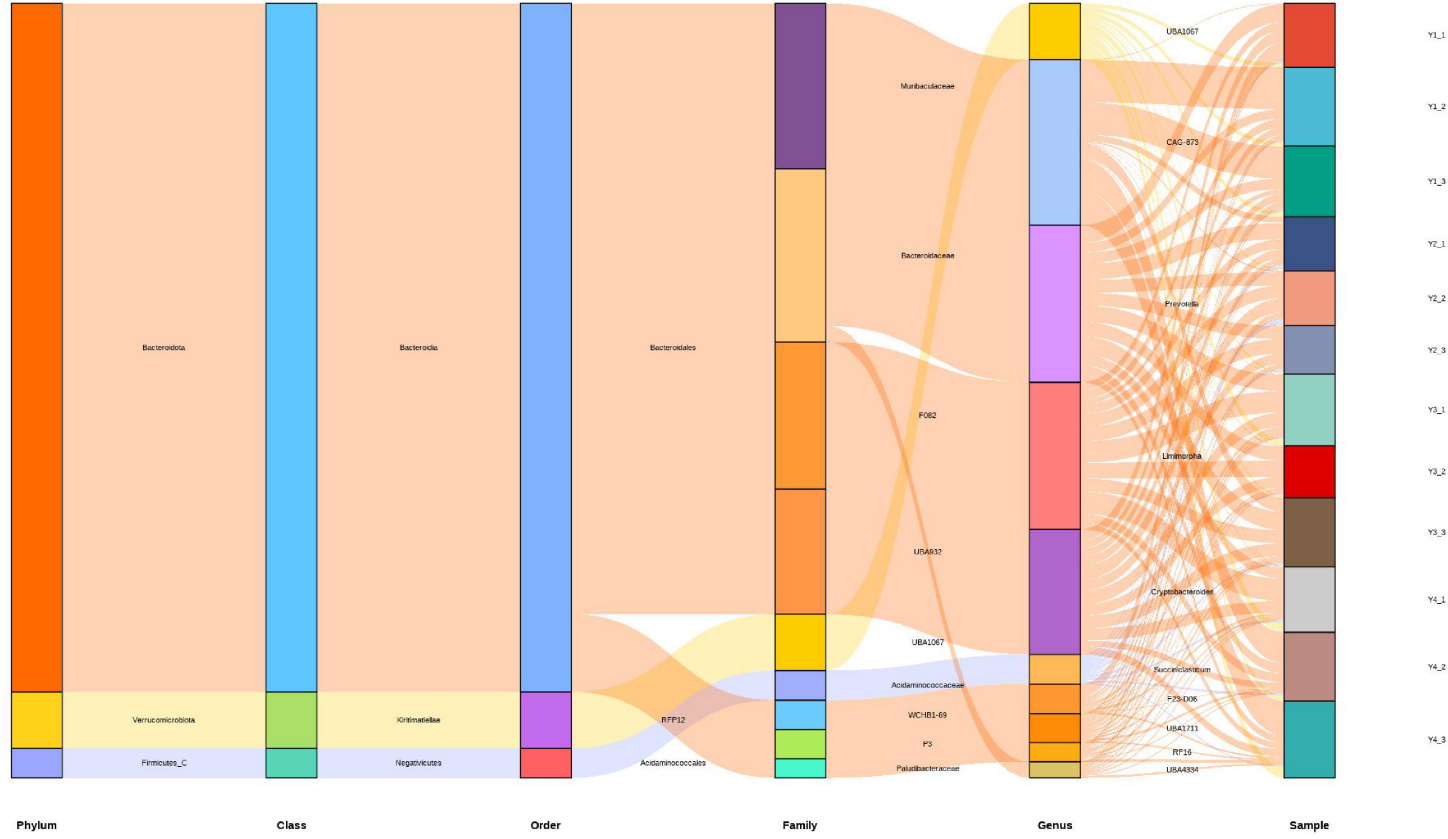
Sankey diagram of rumen microbial taxonomic hierarchy and distribution in sheep fed different LCE levels. (Flows represent phylum-genus connections, with width indicating relative abundance. Y1-Y4 denote 0, 1, 2, and 3% LCE groups, respectively.)

As shown in Figure 5, the Sankey diagram intuitively illustrates the taxonomic hierarchical associations (from phylum to genus) and distribution characteristics of rumen microbiota among different LCE supplementation groups. *Bacteroidetes* and *Firmicutes* were identified as the dominant phyla in the rumen microbial community, with clear taxonomic linkages: *Bacteroidetes* were connected to the family *Prevotellaceae* and its subordinate genus g_Prevotella, while *Firmicutes* was linked to the family Lachnospiraceae and the genus *unidentified_Lachnospiraceae*. Among the experimental groups, the 2% LCE group exhibited distinct flow width characteristics for key microbial taxa, including relatively wider flows for *Firmicutes* and *Limimorpha*, and a more prominent flow for g_Prevotella compared to the 3% LCE group.

#### 3.4.5 Bubble plot analysis of ruminal microbial taxonomic composition and abundance

**FIGURE 6:**
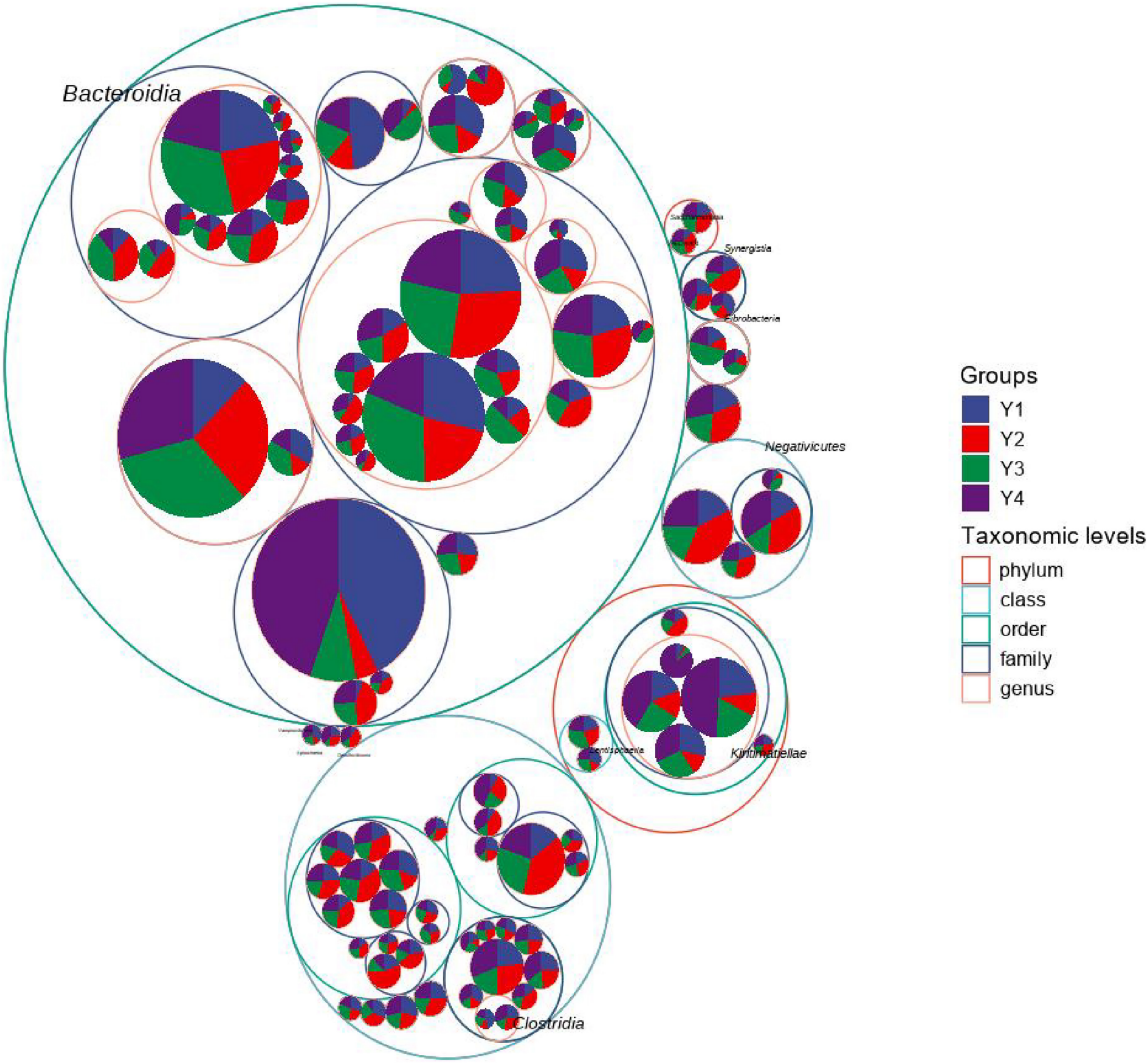
Bubble plot of ruminal microbial taxonomic composition and abundance in sheep fed different LCE levels. Bubble size represents the relative abundance of taxonomic units (phylum to genus), and different colors indicate the four LCE supplementation groups (Y1: 0%, Y2: 1%, Y3: 2%, Y4: 3%).

As presented in Figure 6, the bubble plot visualized the taxonomic composition (from phylum to genus) and relative abundance of rumen microbiota across different LCE supplementation groups. Bubble size corresponds to the relative abundance of each taxonomic unit, with larger bubbles indicating higher abundance. *Bacteroidetes* and *Firmicutes* were the most dominant phyla, with their subordinate taxa (e.g., Bacteroidia, Clostridia, g_Prevotella, and *unidentified_Lachnospiraceae*) showing distinct group-specific distribution patterns. The 2% LCE group (Y3) exhibited larger bubbles for *Firmicutes* and *Limimorpha*, reflecting their higher relative abundances in this group. In contrast, g_Prevotella displayed a smaller bubble in the 3% LCE group (Y4) compared to the 0% (Y1) and 2% (Y3) groups, consistent with the quantitative abundance results.

#### 3.4.6 Analysis of relative abundance of ruminal microbiota at the phylum level

**FIGURE 7:**
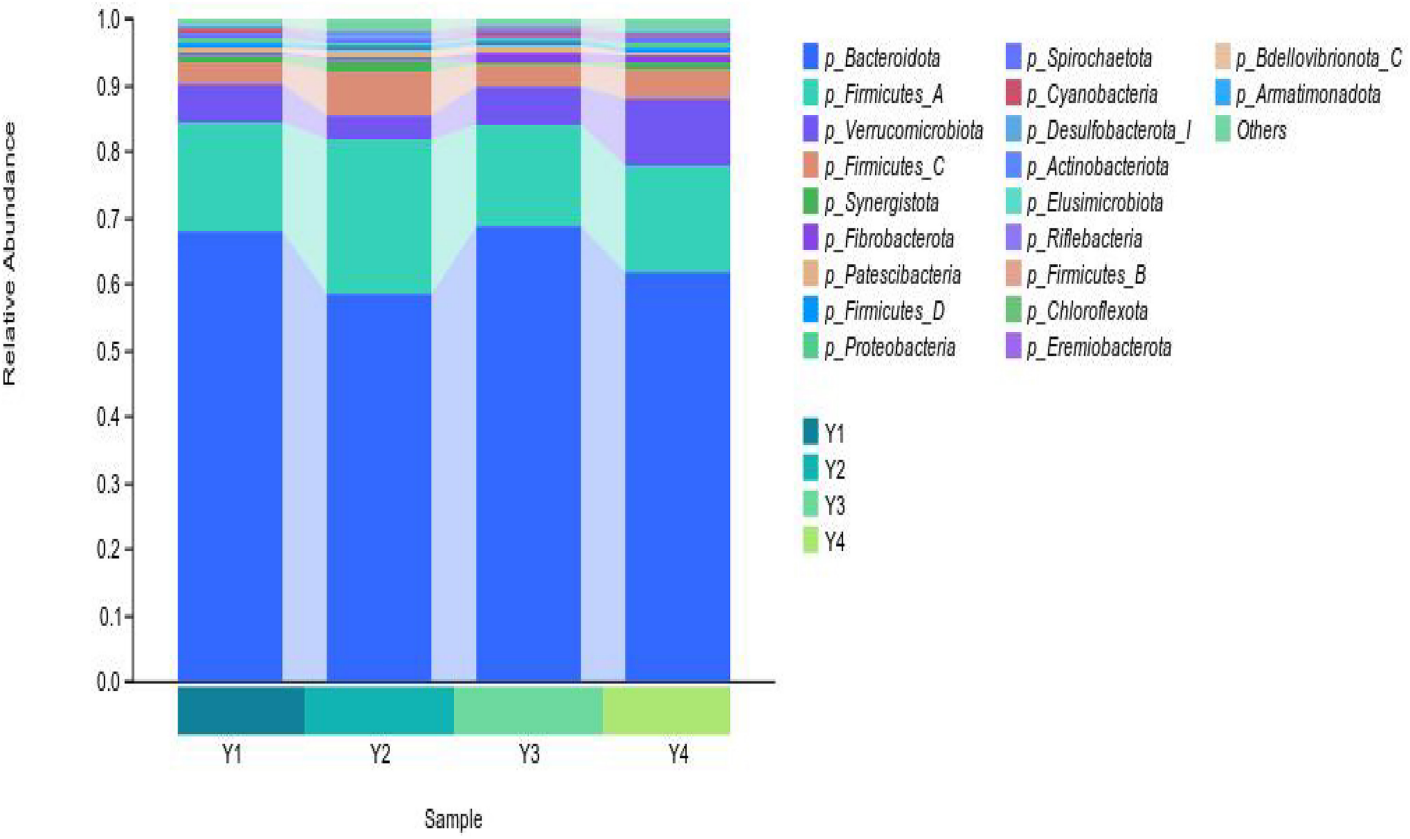
Relative abundance of rumen microbiota at phylum level.

As shown in Figure 7 and Table 6, the relative abundance of *Bacteroidetes* was significantly decreased in the 1% supplementation group, but it increased again in the 2 and 3% groups (*P* < 0.05). In contrast, the abundance of *Firmicutes* was extremely significantly higher in the 2% group (30.55%) compared to all other groups (*P* < 0.05). Furthermore, the Verrucomicrobiota showed a markedly increased abundance in the 3% group (10.17%) relative to the other groups, which ranged approximately between 3.79 and 5.89%. Regarding *Synergistota*, *Fibrobacterota*, *Patescibacteria*, *Proteobacteria*, *Spirochaetota*, *Cyanobacteria*, *Actinobacteria*, and the remaining phyla, no statistically significant differences were observed among the groups (*P* > 0.05).

**TABLE 6:** Effect of adding LCE on the top 10 rumen microbiota at the order level in sheep %.

Items	Groups				*P*-value
	0%	1%	2%	3%	
*Bacteroidetes*	68.07 ± 1.0^a^	58.56 ± 0.8^c^	68.59 ± 3.6^a^	61.76 ± 3.7^b^	0.032
*Firmicutes*	20.10 ± 1.8^b^	19.34 ± 3.0^c^	30.55 ± 1.3^a^	21.25 ± 1.2^b^	0.04
*Verrucomicrobiota*	5.89 ± 0.9	3.79 ± 1.1^c^	5.78 ± 3.3	10.17 ± 2.3^a^	0.045
*Synergistetes*	1.16 ± 1.6	1.75 ± 1.2	0.52 ± 0.5	1.16 ± 0.7	0.786
*Fibrobacteres*	0.61 ± 0.38^b^	0.53 ± 0.2^c^	1.57 ± 1.5^a^	0.61 ± 0.3^b^	0.215
*Patescibacteria*	0.64 ± 0.2	0.91 ± 0.4	0.85 ± 0.1	0.64 ± 0.3	0.632
*Proteobacteria*	0.75 ± 0.4	0.41 ± 0.3	0.57 ± 0.1	0.75 ± 0.2	0.357
*Spirochaetes*	0.58 ± 0.2	0.50 ± 0.2	0.34 ± 0.1	0.58 ± 0.3	0.189
*Cyanobacteria*	0.65 ± 0.3	0.19 ± 0.1	0.56 ± 0.2	0.65 ± 0.1	0.061
*Actinobacteria*	0.18 ± 0.06	0.30 ± 0.11	0.20 ± 0.0	0.18 ± 0.05	0.673
Others	1.37 ± 0.2	2.51 ± 0.1	1.70 ± 0.11	2.04 ± 0.15	0.521

In the same row, values with no letter or the same letter superscripts are not significantly different (*P* > 0.05), whereas those with different small letter superscripts are significantly different (*P* < 0.05).

#### 3.4.7 Analysis of relative abundance of ruminal microbiota at the genus level

**FIGURE 8:**
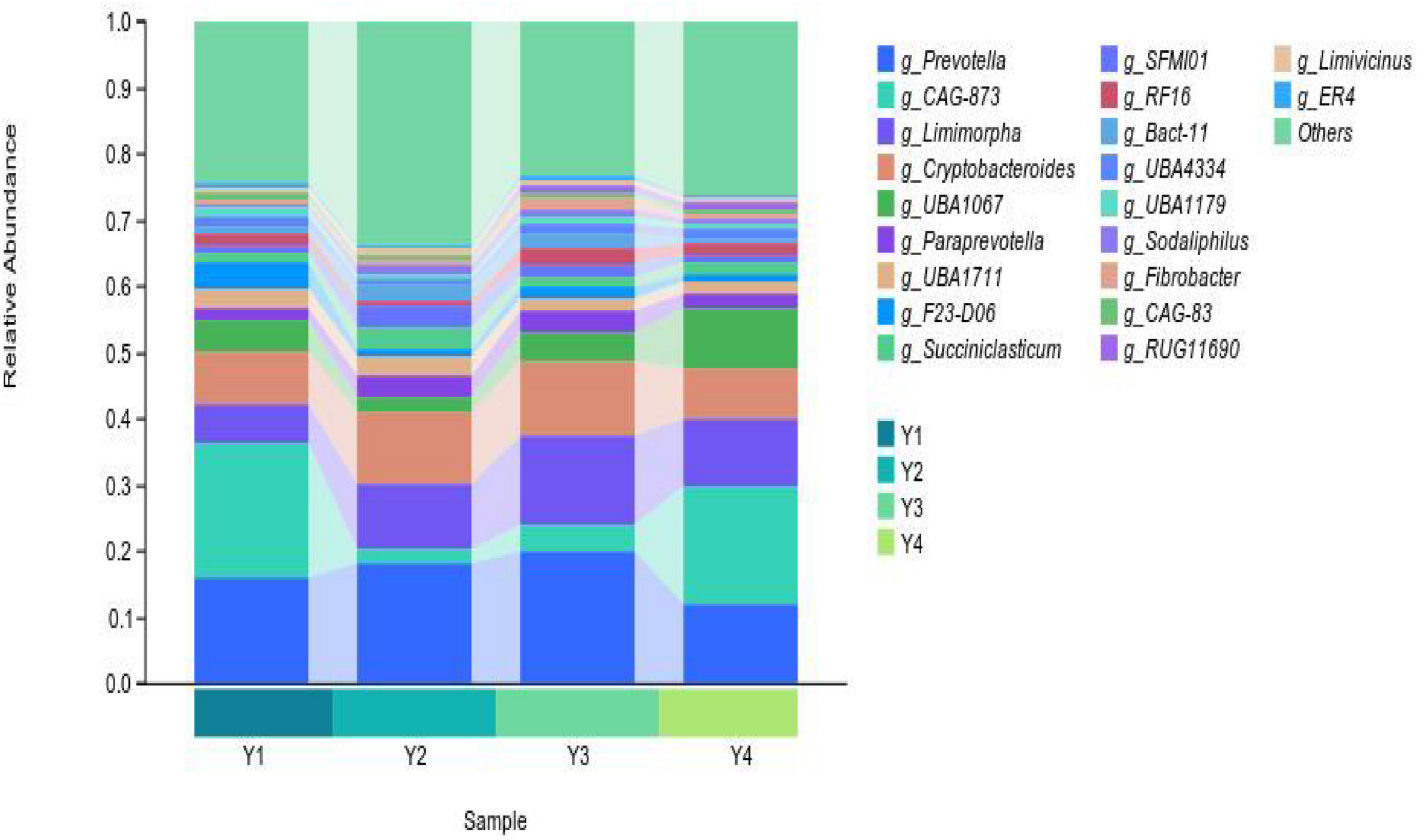
Relative abundance of rumen microbiota at genus level.

Figure 8 and Table 7 reveal that the abundance of *Prevotellaceae* was slightly elevated in the 1 and 2% LCE supplementation groups compared to the control (*P* > 0.05), while it decreased significantly in the 3% supplementation group relative to all other groups (*P* < 0.05). The abundance of *Limimorpha* was significantly elevated in all supplementation groups compared to the control (*P* < 0.05), with the 2% group showing markedly higher abundance than the other three groups (*P* < 0.05). For *unidentified_Bacteroidales*, the abundance increased in the 1 and 2% groups and was significantly greater than in the control and 3% groups (*P* < 0.05). The abundance of *unidentified_Lachnospiraceae* in the 3% supplementation group was significantly higher than that in all other groups (*P* < 0.05).

**TABLE 7:** Effects of added LCE on the genus level of rumen microbiota in sheep (top 10)%.

Items	Groups				*P*-value
	0%	1%	2%	3%	
*Prevotellaceae*	16.11 ± 5.02^b^	18.13 ± 1.45^ab^	19.92 ± 3.13^a^	11.91 ± 2.76^c^	0.028
*CAG-873*	20.16 ± 16.92	2.17 ± 2.55	4.17 ± 5.78	17.93 ± 15.24	0.126
*Limimorpha*	5.84 ± 1.79^c^	10.02 ± 1.83^b^	13.52 ± 3.33^a^	10.07 ± 6.88^b^	0.025
*Unidentified_Bacteroidales*	7.99 ± 1.75^c^	10.71 ± 2.08^b^	11.12 ± 1.0^a^	7.92 ± 2.45^c^	0.041
*Unidentified_Lachnospiraceae*	4.69 ± 0.51^b^	2.44 ± 0.92^c^	4.31 ± 3.09^b^	9.00 ± 2.50^a^	0.005
*Paraprevotella*	2.09 ± 0.56	3.08 ± 1.68	3.24 ± 0.28	2.28 ± 0.67	0.457
*Succinivibrionaceae*	2.77 ± 1.81	2.99 ± 1.15	1.99 ± 1.08	1.5 ± 0.34	0.523
*UBA1711*	1.78 ± 1.41	0.98 ± 0.28	2.46 ± 0.77	1.71 ± 0.60	0.221
*Fibrobacter*	2.60 ± 0.38	2.50 ± 0.19	4.56 ± 1.32	3.76 ± 0.27	0.775
Others	26.07 ± 0.65^b^	26.08 ± 0.32^b^	24.71 ± 0.56^c^	33.92 ± 0.82^a^	0.038

In the same row, values with no letter or the same letter superscripts are not significantly different (*P* > 0.05), whereas those with different small letter superscripts are significantly different (*P* < 0.05).

#### 3.4.8 Random forest analysis of rumen microbial biomarkers

**FIGURE 9:**
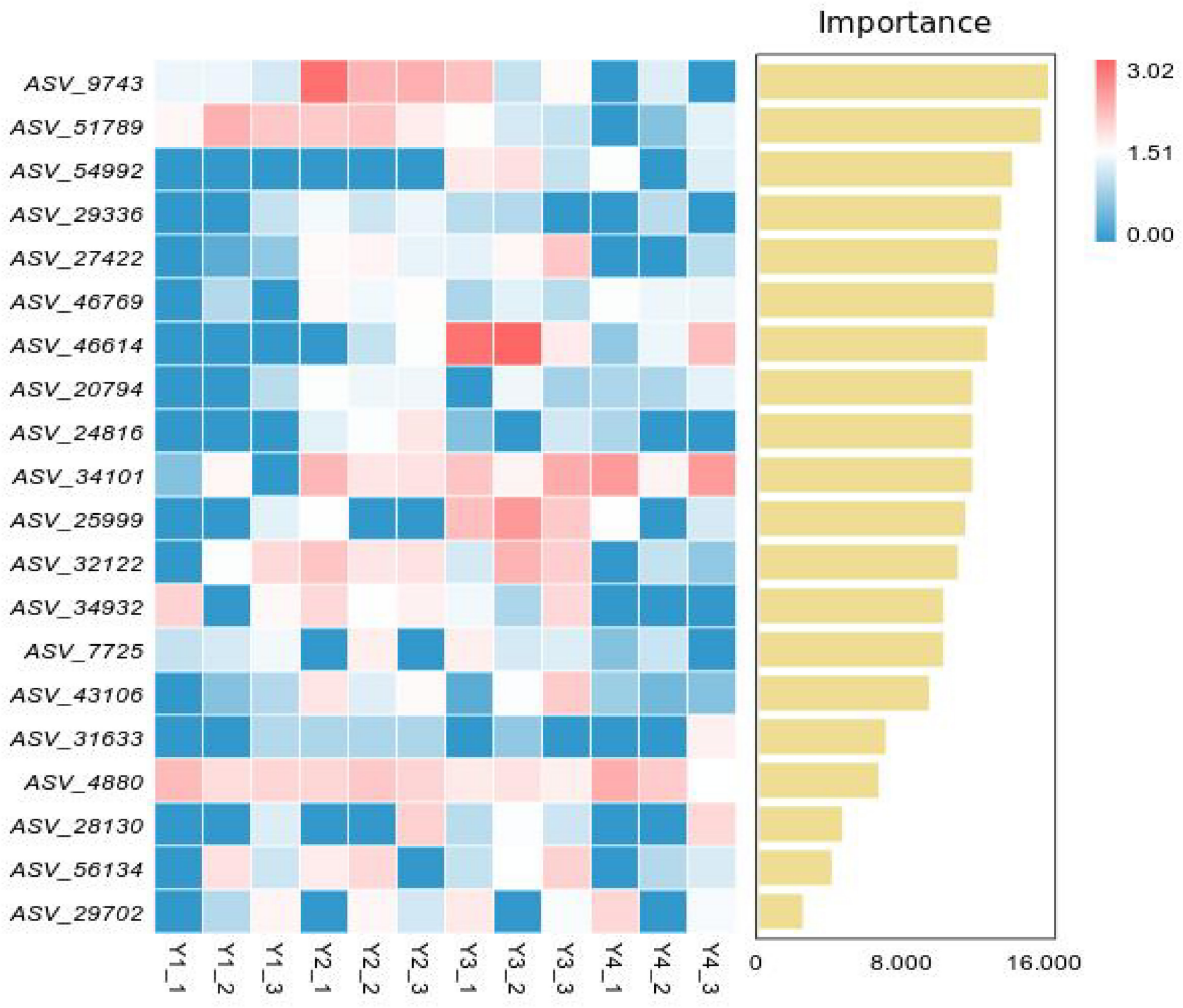
Random forest analysis of rumen microbial biomarkers. The left heatmap shows the relative abundance of key discriminatory ASVs (rows) across samples (columns), with color intensity indicating abundance (red: high abundance; blue: low abundance). The right bar plot represents the importance score of each ASV, reflecting its ability to distinguish between groups. Y1-Y4 denote 0, 1, 2, and 3% LCE groups, respectively.

As shown in Figure 9, random forest analysis was performed to identify the key amplicon sequence variants (ASVs) that discriminated the rumen microbial communities among the four LCE supplementation groups. The importance score (right panel) indicated the contribution of each ASV to the classification of different groups, with longer bars representing higher discriminatory power. Among all identified ASVs, ASV_9743 exhibited the highest importance value, followed by ASV_46614 and ASV_29335, suggesting these ASVs were the core microbial biomarkers for distinguishing the experimental groups. The heatmap visualized the relative abundance distribution of these key ASVs across samples. Specifically, ASV_9743 was highly abundant (dark red) in the 0% LCE group (Y1) but showed low abundance (light color) in the other groups, indicating it was a signature ASV for the control group. In contrast, ASV_46614 was enriched in the 1% LCE group (Y2), and ASV_29335 displayed higher abundance in the 2% LCE group (Y3).

## 4 Discussion

### 4.1 Physiological status in blood

In the current study, LCE supplementation had no significant effect on TP, ALP, and TG (*P* > 0.05), but significantly affected BUN and GLU (*P* < 0.05). The results indicated that LCE had no adverse interference on total protein metabolism, lipid metabolism, or liver function, and the overall metabolic status remained stable. Globulins serve as the primary carriers of immunoglobulins. In the 2% LCE group, GLB concentration was significantly increased (*P* < 0.05), while BUN was decreased, suggesting improved protein utilization efficiency and reduced nitrogen deposition (Zhang et al., 2019). This is consistent with the results of Liang et al., who reported that herbal additives significantly increased serum globulin and reduced BUN concentrations (Liang Y. et al., 2024).

Licorice extract has been proven to enhance antioxidant capacity in animals (Wang et al., 2020). T-AOC and SOD are common indicators for evaluating antioxidant status, and antioxidant enzymes such as GSH-Px play fundamental roles in cellular defense against free radicals (Rodriguez et al., 2004). In the present study, LCE supplementation significantly reduced MDA content (*P* < 0.05) and increased GSH-Px activity (*P* < 0.05)**, indicating improved antioxidant capacity. **SOD and T-AOC showed no significant differences among groups (*P* > 0.05). This result is consistent with Zhang et al. (2015), who reported that adding 0.4% licorice root extract to sheep feed significantly improved antioxidant capacity (Zhang et al., 2015). The enhanced antioxidant capacity may be attributed to the abundant flavonoids in LCE, which can effectively scavenge free radicals (Visavadiya et al., 2009).

Immunoglobulins are important indicators that reflect immune function. In this study, serum IgG, IgM, and IgA showed an upward trend with LCE supplementation, but no significant differences were observed among groups (*P* > 0.05), suggesting that LCE tended to improve the immune status of sheep. This tendency is consistent with the findings of Sun and Pan (2006). Overall, our study demonstrates that 2% LCE effectively improves antioxidant capacity and tends to promote immune function in Karakul sheep.

### 4.2 Rumen pH and volatile fatty acid concentration

The pH value of rumen is a key index to reflect the stability of fermentation (Zhang et al., 2018). However, dietary supplementation with specific amounts of LCE may enhance rumen pH levels, which helps maintain the stability of the rumen environment. Notably, maintaining a pH above 6.0 does not restrict rumen microbial activity (Russell et al., 1992). Meanwhile, rumen NH_3_-N concentration showed a statistically insignificant but decreasing trend with increasing supplementation ratios, suggesting potential improvement in the nitrogen utilization efficiency (Chaturvedi et al., 2015). Volatile fatty acids (VFA), as the primary energy source for ruminants, directly reflect rumen fermentation efficiency and metabolic patterns through their production levels and proportions (Parchami et al., 2024). The TVFA indicate enhanced metabolic activity of rumen microorganisms, indicating more complete carbohydrate fermentation in the diet and providing greater available energy for hosts (Li et al., 2025). In this study, increased TVFA likely resulted from efficient substrate degradation by rumen microbes, altering fermentation metabolism and promoting both fat synthesis and rumen health (Han et al., 2023). Overall, LCE appeared to regulate energy metabolism pathways by optimizing rumen fermentation parameters.

### 4.3 Digestive enzyme activity

In this study, the bioactive components (e.g., flavonoids and polysaccharides) in LCE may have altered the microbial community structure in the rumen and intestines, thereby modifying enzyme activities from different microbial sources (Liang S. et al., 2024). The rumen serves as the primary site for microbial fermentation. The activity of neutral protease in the rumen was significantly reduced (*P* < 0.05), with all treatment groups (1, 2, 3%) showing markedly lower levels than the 0% control group. This is complemented by the increased abundance of *Limimorpha* in the group with 2% LCE (13.52%, *P* < 0.05). This also explains the downward trend in NH_3_-N concentration with LCE addition (7.31–10.13 mg/mL, *P* > 0.05). Notably, the significantly enhanced α-amylase and cellulase activities in the rumen were directly linked to the high abundance of Prevotella (a cellulose-degrading genus) at 2% LCE (19.92%) and the enrichment of unidentified Lachnospiraceae at 3% in the 2% LCE group. These microbial communities provided additional substrates for the VFA production, demonstrating that the LCE addition effectively promotes the proliferation or activity of starch-degrading microorganisms, accelerates the breakdown of dietary fiber, and significantly enhances cellulose-degrading bacteria. This synergistic effect not only improves starch digestion but also enhances crude fiber degradation, consistent with the findings of Liang S. et al. (2024).

In the abomasum, the 1% LCE group showed significantly higher NPT activity compared to the other groups (*P* < 0.05), suggesting that low-dose LCE strongly stimulates protein secretion in the abomasum, though this effect diminishes at high doses. Conversely, the α-amylase activity was significantly reduced (*P* < 0.05). Unlike the rumen, LCE inhibited abomasal amylase activity, indicating its potential impact on the digestive secretion functions in sheep (Cueva et al., 2021). In the duodenum, LCE generally enhances the activity of trypsin, amylase, and cellulase, but shows inconsistent effects on lipase (Wang et al., 2024). In the jejunum, it primarily exhibits inhibitory effects, particularly on amylase and cellulase, which intensifies with increasing dosage. In the ileum, trypsin activity undergoes dramatic fluctuations with higher doses, indicating that high-dose licorice extract severely suppresses protein digestion capacity at the ileal terminal (Wang et al., 2024). Although the rumen protease levels decrease, the increased protease activity in the abomasum (1% group) and duodenum may compensate for an insufficient rumen protein digestion, shifting the site of protein digestion posteriorly. Overall, high-dose supplementation (3%) may exert certain inhibitory effects on the digestive functions in the posterior intestinal segments (jejunum and ileum), requiring precise regulation in practical applications. In summary, LCE regulated digestive enzyme activities in a site-specific manner. It increased cellulase and α-amylase activities in the rumen, enhanced protease activity in the abomasum and duodenum, but slightly inhibited enzyme activities in the jejunum and ileum at high doses. This synergistic pattern effectively improved nutrient digestion and absorption along the gastrointestinal tract.

### 4.4 Diversity of rumen microbes

Rumen pH in the 2% and 3% LCE groups (6.65–6.69) fell within the optimal range (6.5–7.0) for key functional bacteria such as Prevotella and unidentified Lachnospiraceae. This near-optimal pH likely promoted their proliferation and metabolic activity, supporting enhanced fermentation efficiency. In contrast, the control group had a lower pH (6.40–6.42), which may inhibit the activity of some acid-producing bacteria, resulting in a significantly lower VFA yield (89.18 mmol/L) compared with the supplemented groups. These results indicate that LCE not only affects fermentation by directly regulating the microbial composition, but also optimizes the rumen internal environment to provide a guarantee for the functional bacteria to exert their effects. The Alpha diversity index serves as a key metric for assessing both richness and evenness in rumen microbial communities (Liu et al., 2024). Our study demonstrated that compared to the control group (0%), the 1 and 2% LCE supplementation groups exhibited significantly elevated Alpha diversity indices (including Chao1, Shannon, Simpson, and observed species counts) (*P* < 0.05), indicating that these doses of LCE effectively increased both the taxonomic diversity and distribution uniformity of rumen microorganisms. A highly diverse rumen microbiota can more efficiently break down various feed components and adapt to dietary changes, thereby maintaining stable host productivity (Liu et al., 2024). However, it is noteworthy that the diversity index declined in the 3% supplementation group, which may be attributed to antimicrobial activity of certain components that inhibited the growth of specific microbial species, ultimately leading to reduced diversity.

At the phylum level, *Bacteroidetes* and Firmicutes are dominant microbial groups, collectively accounting for over 85% of the total proportion, consistent with the existing research reports (Gruninger et al., 2019). The addition of LCE significantly altered their relative abundance (*P* < 0.05). In the 2% supplementation group, *Firmicutes* proportionally increased to 30.55%, while *Bacteroidetes* decreased to 58.56%. Firmicutes contain numerous important cellulose-degrading and starch-degrading bacteria, whose elevated proportion is often associated with improved energy acquisition efficiency (Cholewińska et al., 2021). This finding aligns with the previous data showing significant increases in TVFA in the 1 and 2% LCE supplementation groups, suggesting that enhanced TVFA production may directly result from increased metabolic activity in Firmicutes and other fermentative microbiota. At the genus level, *Fibrobacter* showed higher trends than the control group in both 2 and 3% LCE supplementation groups, corroborating previous findings of significantly enhanced cellulase activity at these concentrations. Collectively, these results demonstrate that LCE helps improve the digestion of fibrous substances (Wang et al., 2024). The *Prevotellaceae* family demonstrated the highest relative abundance in the 2% LCE group (19.9%), while showing the lowest abundance in the 3% LCE group (11.9%) which was highly consistent with the trend of acetic acid (AA) concentration. The 2% LCE group showed AA levels reaching 71.5 mmol/L (significantly higher than the control group’s 54 mmol/L), while the 3% LCE group saw AA levels drop to 61.9 mmol/L. It is hypothesized that LCE may moderately stimulate Prevotella’s polysaccharide degradation activity (e.g., cellulose and hemicellulose breakdown) at low concentrations (1–2%), promoting the production of acetate precursors like pyruvate. However, at high concentrations (3%), certain active components in LCE might inhibit Prevotella, leading to a simultaneous decrease in both Prevotella abundance and acetate yield (Li et al., 2021). In conclusion, as a plant-based additive, LCE can significantly reshape the rum microbial ecosystem, with its efficacy exhibiting clear dose-dependent characteristics. The 1 and 2% LCE addition levels demonstrated the most positive regulatory effects across most indicators.

Most previous studies used purified licorice extracts, whereas this study used industrial licorice crude extract (LCE). LCE retained functional components such as flavonoids and polysaccharides, but with lower cost and higher feasibility for ruminant production. This provides a new strategy for the high-value utilization of industrial by-products. In conclusion, as a plant-based additive, LCE can significantly reshape the rum microbial ecosystem, with its efficacy exhibiting clear dose-dependent characteristics.

## 5 Conclusion

This study demonstrates that dietary supplementation with licorice crude extract (LCE) comprehensively improves the antioxidant capacity, immune function, rumen fermentation, gastrointestinal digestive enzyme activities, and rumen microbial diversity of Karakul sheep. Among all doses, dietary supplementation with 2% LCE most comprehensively improved antioxidant capacity, immune function, rumen fermentation, microbial diversity, and gastrointestinal enzyme activities in Karakul sheep. Although some indexes showed fluctuating responses at 1% or 3% LCE, the 2% level achieved the best balance and most stable beneficial effects. Thus, 2% LCE is recommended as the optimal inclusion level for sheep feeding. As a low-cost industrial by-product, LCE retains the bioactive functions of licorice and can be used as an effective feed additive to promote sheep health and production efficiency. While this work establishes a theoretical basis for developing LCE as a ruminant feed additive, its specific mechanisms require further investigation, and precise regulatory strategies for evaluations are critical for its practical application.

## Data Availability

The raw sequence data of rumen microbiota are available in the NGDC Genome Sequence Archive (https://ngdc.cncb.ac.cn/gsa), under accession CRA034220.
